# Induction of stromule formation by extracellular sucrose and glucose in epidermal leaf tissue of *Arabidopsis thaliana*

**DOI:** 10.1186/1471-2229-11-115

**Published:** 2011-08-16

**Authors:** Martin Hartmut Schattat, Ralf Bernd Klösgen

**Affiliations:** 1 Laboratory of Plant Development and Interactions; Department of Molecular and Cellular Biology; University of Guelph; Guelph, ON Canada; 2 Institute of Biology - Plant Physiology, Martin-Luther-University Halle-Wittenberg, Weinbergweg 10, 06120 Halle (Saale), Germany

## Abstract

**Background:**

Stromules are dynamic tubular structures emerging from the surface of plastids that are filled with stroma. Despite considerable progress in understanding the importance of certain cytoskeleton elements and motor proteins for stromule maintenance, their function within the plant cell is still unknown. It has been suggested that stromules facilitate the exchange of metabolites and/or signals between plastids and other cell compartments by increasing the cytosolically exposed plastid surface area but experimental evidence for the involvement of stromules in metabolic processes is not available. The frequent occurrence of stromules in both sink tissues and heterotrophic cell cultures suggests that the presence of carbohydrates in the extracellular space is a possible trigger of stromule formation. We have examined this hypothesis with induction experiments using the upper epidermis from rosette leaves of *Arabidopsis thaliana *as a model system.

**Results:**

We found that the stromule frequency rises significantly if either sucrose or glucose is applied to the apoplast by vacuum infiltration. In contrast, neither fructose nor sorbitol or mannitol are capable of inducing stromule formation which rules out the hypothesis that stromule induction is merely the result of changes in the osmotic conditions. Stromule formation depends on translational activity in the cytosol, whereas protein synthesis within the plastids is not required. Lastly, stromule induction is not restricted to the plastids of the upper epidermis but is similarly observed also with chloroplasts of the palisade parenchyma.

**Conclusions:**

The establishment of an experimental system allowing the reproducible induction of stromules by vacuum infiltration of leaf tissue provides a suitable tool for the systematic analysis of conditions and requirements leading to the formation of these dynamic organelle structures. The applicability of the approach is demonstrated here by analyzing the influence of apoplastic sugar solutions on stromule formation. We found that only a subset of sugars generated in the primary metabolism of plants induce stromule formation, which is furthermore dependent on cytosolic translational activity. This suggests regulation of stromule formation by sugar sensing mechanisms and a possible role of stromules in carbohydrate metabolism and metabolite exchange.

## Background

Stromules (stroma filled tubules) [1] are protrusions of the plastid envelope with a diameter of usually less than 1 μm [2]. These filament-like structures are highly dynamic and can extend and retract within seconds [3]. Although tubules extending from the plastid surface had been described in a monograph about plastids in 1908 (see [4]), their significance and morphological relevance was recognized only after development and improvement of suitable fluorescence microscopy techniques. In particular, the generation of transgenic plants expressing chimeric proteins consisting of green fluorescent protein (GFP) fused to chloroplast targeting transit peptides allowed the first detailed analysis demonstrating the presence of stromal proteins within these structures [1]. Over the past years, stromules were found in a variety of vascular plant species, non-vascular plant species and green algae (as summarized in [5]) which suggests evolutionary conservation of these structures and implies that they might play an important role in all members of the *Viridiplantae*. Despite significant progress in understanding the importance of certain cytoskeleton elements and motor proteins for stromule maintenance [6-8], the function of stromules remains elusive. One way to approximate their role in plant cells is to search for physiological conditions which lead to the induction of stromules.

Stromules are found at relatively high frequency, for example, in sink tissues like ripening tomatoes [9], in leaf samples placed on sucrose-rich medium as well as in BY2 cell cultures [10]. In all these instances, the cells showing high stromule frequency are exposed to a relatively high concentration of carbohydrates which suggests a link between the presence of sugars and stromule formation. Here, we have tried to elucidate this potential correlation by measuring the influence of exposure to different sugar solutions on stromule frequency in a model plant tissue, notably the upper leaf epidermis of *Arabidopsis thaliana*.

We found in our experiments that stromule formation is strongly induced in epidermal plastids after application of sucrose and glucose. The specificity of this induction is confirmed by the inability of either fructose, sorbitol and mannitol to induce the same reactions. Furthermore, stromule induction by sucrose and glucose is not restricted to epidermal plastids but can likewise be observed in chloroplasts of the palisade parenchyma.

## Results

### Upper leaf epidermis as suitable model tissue for stromule induction

**Figure 1:**
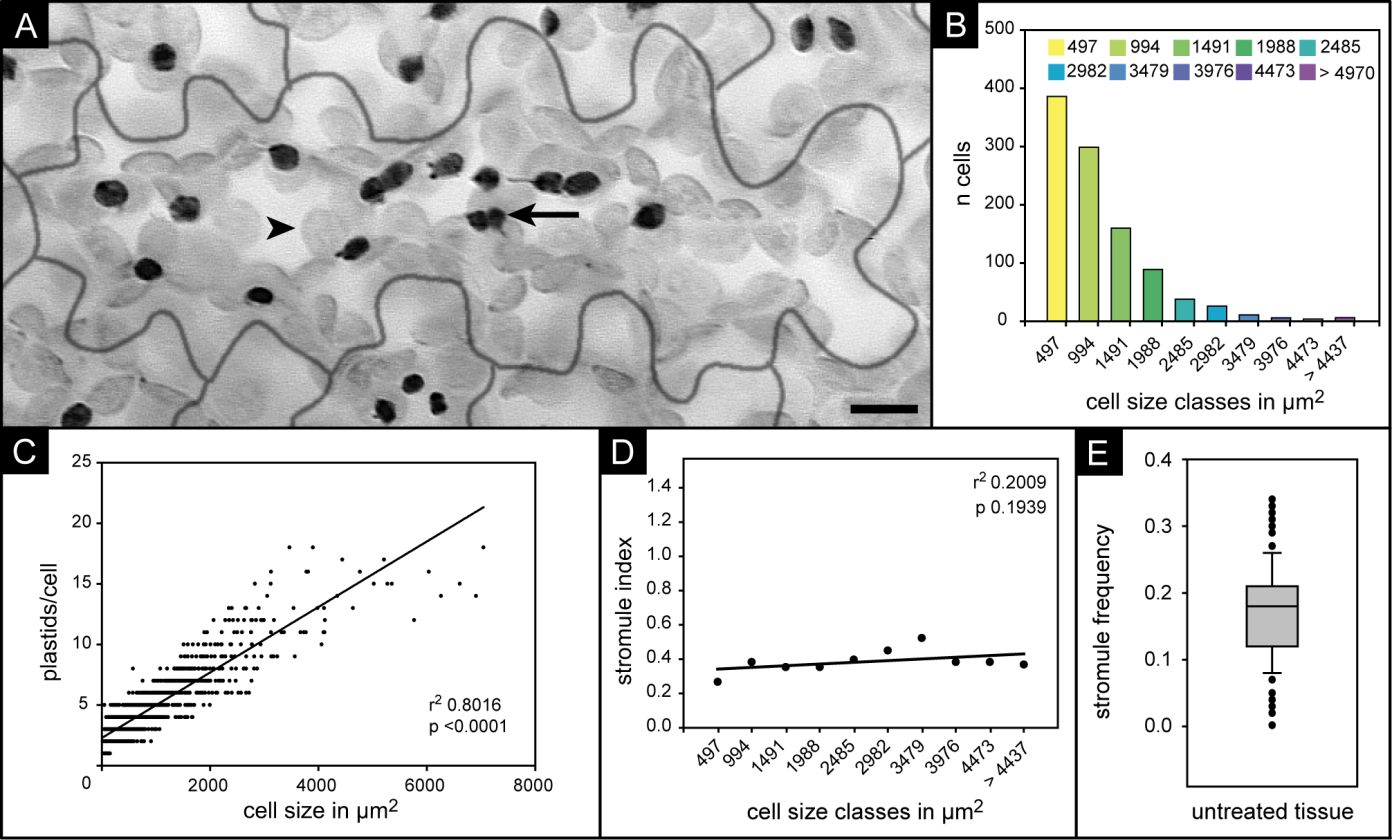
**characteristics of the upper leaf epidermis**. **A**) In 'Stacked' images of upper epidermal cells of the *Arabidopsis thaliana *line FNR/EGFP-7-4, all plastids in a given cell are visible (cell boundaries given as grey line). For better display of stromules, the image has been gray scaled and inverted. Therefore, epidermal plastids are visible in dark grey (arrow) and the larger plastids from the palisade parenchyma appear light grey (arrowhead). Size bar corresponds to 10 μm. **B**) Histogram of cell size in the upper epidermis, illustrating the huge variety of cell sizes and the predominance of small cells in this tissue. Values given on x-axes are upper limits of size classes. The visible surface area, defined by the lateral cell walls, was used as a measure of cell size, (see A). **C**) Scatter plot and linear regression line of plastid number vs. cell size showing the strong linear correlation between both variables (r^2 ^0.8016; p value < 0.0001). This underlines that cells of the epidermis can be very different in cell size and plastid number. **D**) Plot and linear regression line of stromule frequency vs. cell size class suggesting no significant correlation between the two parameters. Because of unequal class size, ' > 44376' has not been considered in the regression test. **E**) Box plot of stromule frequency found in 109 independent samples of untreated upper leaf epidermis. Specific parameters: maximum = 0.34, 90% percentile = 0.26, 75% percentile = 0.21, median 0.18, 25% percentile = 0.13, 10% percentile = 0.08, minimum 0.02.

For the intended experiments, a suitable model system is required that allows both controlled exposure to sugar solutions and easy analysis of sufficiently high numbers of plastids. We chose *Arabidopsis thaliana *as model plant, notably a transgenic line (FNR/EGFP-7-4) which constitutively expresses FNR/EGFP, a chimeric protein composed of the chloroplast targeting transit peptide of ferredoxin-NADP-oxidoreductase (FNR) fused to EGFP, a derivative of green fluorescent protein with enhanced fluorescence properties [11,12]. Due to the resulting green fluorescent stroma, plastids and stromules can easily be visualized in this transgenic line by conventional epifluorescence microscopy (Figure 1A). In order to avoid exposure of plant material to external sugars during cultivation which might interfere with the intended sugar induction experiments soil grown plants were used. The upper epidermal tissue of rosette leaves was chosen for these studies because it facilitates relatively easy data acquisition. Due to the flat shape of these cells and the comparably low number of plastids (usually below 20), all plastids and stromules within a given cell can be monitored by epifluorescence microscopy in a z-stack consisting of less than 20 focal planes (Figure 1A). Furthermore, its surface is not as textured as that of the lower epidermis with its emersed vascular veins, which would impede microscopic imaging.

### Stromule frequency is not influenced by cell size

To identify changes in the chosen model tissue a suitable stromule parameter is needed. Stromule frequency (SF), i.e. the proportion of plastids showing at least one stromule, has been previously used for quantification of changes induced by experimental treatments to epidermal cells of *Nicotiana benthamiana *[13] and occurring during ripening of tomato fruit [9]. Because SF is a proportion, Waters and colleagues [9] introduced the 'stromule index' for statistical analysis and graphical display, which represents the arcsin transformed SF (arcsin √ x, where as × is the proportion). SF, as it has been used before, is based on the comparison of SF estimated for individual cells. This necessitates the cell context to be considered and requires imaging of plastids and cell boundaries. Not having to consider the cell context would allow for streamlined imaging and data analysis but would presuppose that cells of different size do not differ in SF. Our measurement of the size of 1023 cells from 3 leaves (as measure of cell size the surface area bound by the lateral cell wall was used) shows that the upper epidermis of *Arabidopsis thaliana *is composed of cells of very different size (ranging in our measurements from 15 μm^2 ^to 7044 μm^2^) with small cells dominating the epidermis by number (Figure 1B and additional file 1 panel A). As illustrated in Figure 1C, the number of plastids per cell (ranging in our measurements from 1 to 20) is positively correlated with cell size (coefficient of determination r^2 ^0.8016 and p < 0.0001). In order to test if stromule frequency changes with cell size, cells were pooled into size classes incrementing by 497 μm^2^. The respective SF was calculated by dividing the total number of plastids exhibiting stromules by the number of all plastids within a given size class. As Figure 1D shows, a correlation of cell size and SF is unlikely in this tissue (r^2 ^0.009; p 0.1939; because of unequal size of size class ' > 4437' this class has not been considered for regression analysis). Taking this into account, stromule frequency in the upper epidermis can be estimated without considering the cell context, which makes imaging cell boundaries unnecessary.

### Stromule frequency varies in untreated leaves

In order to determine the variability of SF in untreated tissue, SF of 109 leaves from different plants was estimated. The box plot shown in Figure 1E illustrates that 50% of the samples had a SF ranging from 0.13 to 0.23 with a median of 0.18, while in few cases (< 20 plants) leaves showed markedly deviating frequencies (< 0.09 or > 0.27). By using different leaves for different time points in a time course experiment, such variation could mask potential inducing effects. Therefore, we used leaf squares from a single leaf for a given time course experiment.

### Stromule formation is induced by extracellular sucrose

The most prominent transport sugar present in the phloem sap of plants is sucrose [14] which is usually unloaded from the phloem into the apoplast of the sink tissue. Sucrose is also the main carbon source in media used for plant tissue culture. In both types of cells, stromules have been observed in high abundance [9,10] which suggests that the presence of sucrose in the apoplast of plant cells might support stromule formation. In order to experimentally test this hypothesis, we infiltrated leaves of *Arabidopsis thaliana *with a buffer solution (APW) supplemented with 40 mM sucrose, a concentration routinely used in *Arabidopsis thaliana *tissue culture medium.

**Figure 2:**
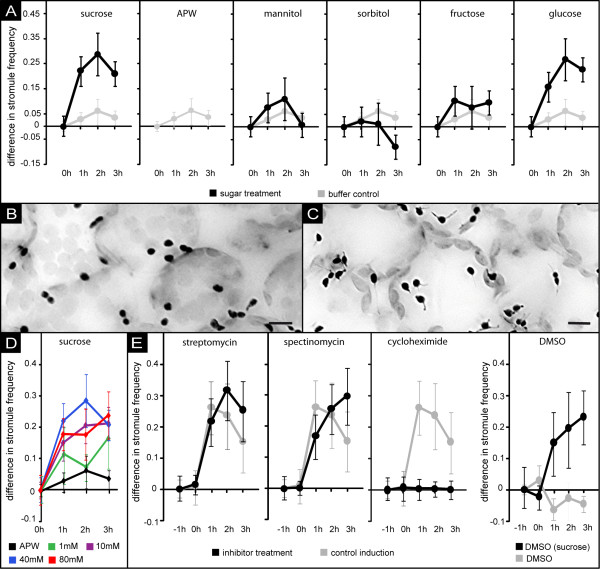
**change in stromule frequency in response to sugar exposure**. **A**) Line plots illustrating changes in SF over time after vacuum infiltration of either 40 mM sugar solution (sucrose, mannitol, sorbitol, fructose or glucose; depicted as black line), or buffer control (APW, depicted as grey line). Error bars represent the 99% confidence intervals. For better comparison, values of the buffer control were plotted along with the sugar treatments. For absolute SF values see additional file 1C. **B-C**) 'Stacked' and inverted epifluorescence images showing leaf epidermal plastids (black) either 0 h (B) or 2 h (C) after infiltration of 40 mM glucose solution. Note the significantly higher number of plastids having stromules in the image taken at 2 h (C). Size bar corresponds to 10 μm. **D**) Line plot depicting changes of stromule frequency induced by different sucrose concentrations (1 mM, 10 mM, 40 mM, and 80 mM) as well as by the buffer control (APW). The plots show that increase of sucrose concentration above 40 mM does not result in stronger stromule induction. Error bars represent the 99% confidence intervals. Values for 40 mM and APW have been taken from previously shown experiments (A). **E**) Line plots with 99% confidence intervals showing the time-course of increase in SF in the presence or absence of translational inhibitors. Leaf samples were pretreated for 1 h (-1 h to 0 h) with APW supplemented with either cycloheximide, DMSO, streptomycin, or spectinomycin. At time point "0 h", all buffers were additionally supplemented with 40 mM sucrose and incubated for additional 3 h. For further details see the legend to A of the same figure.

We used vacuum infiltration of the leaf tissue to ensure fast and uniform exposure of cells to the solution. At distinct time points (0, 60, 120, and 180 min), single samples of a given leaf were analyzed independently from each other by fluorescence microscopy (for a scheme of the infiltration and incubation procedure see additional file 1 panel B). After image processing, the number of plastids with or without stromules was counted for each sample allowing determination of SF. Each treatment was carried out three times. The resulting changes in SF are shown as mean values along with the 99% confidence intervals in Figure 2A (for absolute values of stromule index and stromule frequency see additional file 1 panel C and D). As illustrated by the graphs in Figure 2A and the microscopic images depicted in Figure 2B and 2C, SF increases dramatically during the first 60 min of exposure to 40 mM sucrose (for complete image series see additional file 2). After 120 min, maximal SF is observed and further exposure to the sucrose solution leads to slowly decreasing SF. Control leaf samples were treated as described above except that APW lacking sucrose was used for infiltration. In these samples, only marginal increase in SF compared to non-infiltrated samples was observed (Figure 2A) demonstrating that the presence of sucrose, and not the vacuum treatment of the leaf tissue, is responsible for stromule induction.

Like other sucrose-induced physiological reactions, e.g. anthocyanin accumulation in *Arabidopsis thaliana *seedlings or alpha-amylase induction in barley embryos [15,16], the increase in SF is concentration dependent. Infiltrating tissue samples with solutions of 1 mM, 10 mM or 40 mM sucrose suggests correlation of SF and sucrose concentration of the infiltration medium (Figure 2D; for absolute values of stromule index and stromule frequency see additional file 1 panel E and F). However, a further increase in sucrose concentration (> 40 mM) did not lead to an additional increase in SF suggesting a kind of saturation effect. Furthermore, we have never observed SF exceeding 60%, i.e. even under "optimal" inducing conditions approximately 40% of all plastids in a sample are yet devoid of visible stromules.

### Induction of stromule formation is not an osmotic effect

In order to elucidate whether sucrose induction of stromule formation is merely an osmotic effect, the experiments were repeated with solutions of mannitol and sorbitol, which are both not part of the primary carbon metabolism and are routinely used to apply osmotic stress [17-20]. In neither case did vacuum infiltration result in significant increase in SF (Figure 2A). Instead, infiltration of sorbitol led even to a mild inhibition of stromule formation demonstrating that changes in osmotic conditions cannot be the reason for the stromule induction observed in the presence of sucrose. This suggests that stromule formation can be elicited specifically by sugars present during primary carbon metabolism.

However, not even all of these sugars are capable of inducing stromule formation. If sucrose is replaced by either glucose or fructose in the infiltration experiments only glucose was able to induce stromule formation, whereas fructose treatment did not lead to any change in SF (Figure 2A). Although the process is not well understood, it is widely accepted (based on supporting experimental evidence, summarized in [21]) that extracellular fructose generated by sucrose-cleavage in the apoplast is imported into the cell. Considering that intracellular fructose can be converted to phosphorylated glucose, stromule induction is probably not caused by an overall increase in cell metabolic activity but likely depends on specific metabolic and/or signaling pathways.

### Stromule formation requires *de novo *protein synthesis in the cytosol

The apparent influence of metabolic activity on stromule formation was further analyzed by sucrose induction experiments performed in the presence of inhibitors of protein biosynthesis. If the sucrose solution is additionally supplemented with cycloheximide (CHX), which inhibits the activity of 80S ribosomes and thus is used to prevent translation in the cytosol [22,23], we observed complete inhibition of stromule formation (Figure 2E). In contrast, control experiments performed with sucrose solutions supplemented with 0.03% DMSO, the solvent of CHX, did not show any inhibitory or inducing effect (Figure 2E) confirming the specificity of this reaction. On the other hand, neither streptomycin nor spectinomycin, which are used to prevent translation within plastids by inhibition of the 70S ribosomes [22,24-26], affected sucrose-triggered stromule induction (Figure 2E). This indicates that *de novo *synthesis of nuclear encoded proteins but not of those encoded by the plastid genome is required to mediate the signal from apoplastic sucrose accumulation to stromule formation.

### Sucrose-dependent stromule formation is observed also in the palisade parenchyma

**Figure 3:**
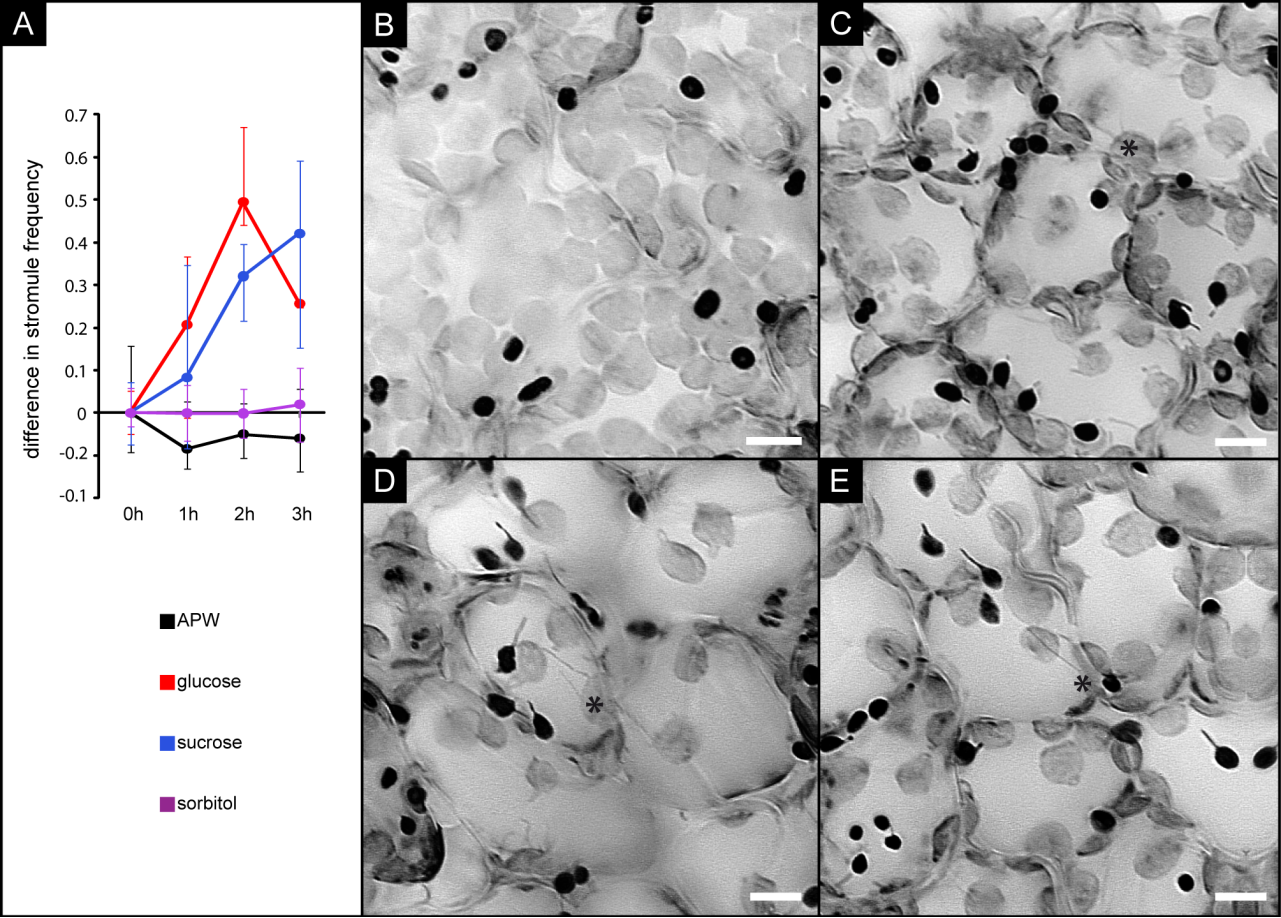
**response of palisade parenchyma plastids to sugar exposure**. **A**) Increase of stromule frequency in mesophyll cells after vacuum infiltration of either APW or APW supplemented with 40 mM glucose, 40 mM sucrose, or 40 mM sorbitol. Error bars indicate the 99% confidence intervals. For absolute values of SF see additional file 2 panel B. **B - E) **'Stacked' and inverted epifluorescence images showing leaf tissue either at 0 h (B), 1 h (C), 2 h (D) or 3 h (E) after infiltration of 40 mM glucose solution. The plastids of epidermal cells appear in dark, while the larger mesophyll chloroplasts appear brighter. Note the increasing proportion of plastids in both tissues that form stromules. The asterisk highlights mesophyll chloroplasts with stromules. Size bar corresponds to 10 μm.

In order to examine if fully developed chloroplasts are also competent for stromule formation following sucrose and glucose treatment, the image stacks obtained in the experiments shown in Figure 2A were additionally screened for the presence of palisade parenchyma cells. This photosynthetically active tissue carries fully developed chloroplasts. Indeed, we detected in the images taken for sucrose, glucose, APW and sorbitol treatments not only sufficient amounts of chloroplasts but found that those chloroplasts showed stromule induction characteristics indistinguishable from those of the epidermal plastids. While solutions of sucrose and glucose led to pronounced induction of stromule formation, neither sorbitol nor the buffer control had any stimulatory effect (Figure 3A; for absolute values of stromule index and stromule frequency see additional file 3 panel A and B). Even the maximal SF determined for epidermal cells after sucrose induction (60% in single treatments, for the mean of absolute SF values see additional file 1 panel D and F and compare with additional file 3 panel B) was observed for the chloroplasts of the photosynthetically active cells. It should be noted though that the detection of stromules in these cells was complicated by the dense packing of most chloroplasts. Furthermore, many chloroplasts of parenchyma cells in the field of view were not captured in the images and could thus not be considered for estimating stromule frequency. Both factors might have contributed to the relatively large confidence intervals. However, our data still clearly demonstrate that stromule induction by selected sugars is not restricted to the plastids found in the upper leaf epidermis and suggests that the mechanism leading to stromule formation is conserved among diverse plastid types.

## Discussion

In the present study, we aimed to establish an experimental system facilitating the reproducible induction of stromule formation in living plant tissue in order to make these enigmatic structures better accessible to systematic investigation. A possible connection between extracellular sugars and stromule formation has been suggested by several reports concerning high stromule frequency in heterotrophic cell cultures and sink tissues. Using the upper leaf epidermis of a transgenic *Arabidopsis thaliana *line harboring green fluorescent plastid stroma as model tissue, we addressed the influence of extracellular sugars on stromule formation.

### Stromule formation is specifically induced by sucrose and glucose

We found that formation of stromules is specifically induced only by a subset of sugars generated in plants. While vacuum infiltration of either sucrose or glucose leads to a significant increase in SF, an inducing effect of fructose or mannitol cannot be observed. Infiltration of sorbitol leads even to a mild inhibition of stromule formation. Thus, our data suggest that stromule formation is most likely due to neither osmotic effects nor the result of the presence of metabolizable sugars in general. Instead, it seems that specific signaling pathways involving sucrose and/or glucose play a role in the induction process.

The role of sucrose in signal transduction is difficult to evaluate despite the fact that there is strong evidence for sucrose-specific intracellular and extracellular sensing mechanisms operating in plants [15,27]. Since sucrose is efficiently cleaved into fructose and glucose, both in the apoplast and in the cytosol by invertases and sucrose synthase, the signaling function of sucrose is difficult to distinguish from that of its cleavage products - glucose or UDP-glucose. Glucose sensing, on the other hand, is already understood in some detail. In particular, the intracellular enzyme hexokinase1 (HXK1) has been identified as a key player in this process. Beside its enzymatic activity, HXK1 is an important glucose sensor. Isoforms of this enzyme are present within plastids as well as associated with mitochondria. The latter isoform is also found in the nucleus where it is part of a protein complex involved in gene regulation [28]. In addition to HXK1, further potential glucose sensors have been reported, which alternatively or additionally might be involved in glucose induced stromule formation [27].

At this stage, it is not known if sucrose and glucose can act as independent signals for stromule formation or if the sucrose induction observed is caused by the release of glucose after sucrose cleavage. Likewise, the question remains to be answered as to whether the stromule inducing signal is sensed extracellularly or intracellularly.

It should be kept in mind that changes in extracellular sugar levels might not only influence the carbohydrate metabolism of a cell but may be a cause of stress for the plant cells potentially leading to the induction of stress signaling pathways [29]. Further experimental evidence is therefore required to substantiate the presumed interdependence of stromule formation and carbohydrate metabolism. Hence, our next experiments will address the question if glucose and sucrose generate independent stromule inducing signals and if internal changes in sugar levels are sufficient to change stromule frequency (making use, for example, of non-metabolizable glucose and sucrose analogues as well as mutants with altered intracellular sucrose and glucose levels).

### Stromules may support metabolite exchange

Although several possible functions for stromules have been suggested and discussed [5], the final role of stromules in plant cells remains still enigmatic. The observation of stromules or other envelope protrusions being in direct contact with mitochondria and peroxisomes led to the suggestion that formation of envelope protrusions, like stromules, supports photorespiration [30-33] by increasing the interactive surface between the organelles and, in turn, facilitating efficient metabolite exchange. However, experimental evidence that these organellar connections become more frequent under photorespiratory conditions, which would support this assumption, is yet missing.

Alternatively, the increase in interactive plastid surface by stromule formation may have more general consequences on the interaction of plastids with the cytosol or other organelles, which might be particularly relevant under conditions of increased demand of metabolite import or export across the plastid envelope membranes [2,5]. Indeed, our results demonstrating stromule induction by sucrose and glucose seem to support this hypothesis. Apoplastic glucose and/or sucrose are particularly prominent in sink tissue and heterotrophic cell cultures. The non- or less-photosynthetically active plastids of these cells import large amounts of glucose-6 phosphate from the cytosol in order to generate the ATP and NADPH needed to fulfill their metabolic functions, which in turn originates from extracellular sucrose or glucose pools. On the other hand, triose phosphates, which are simultaneously produced in this process, are exported back into the cytosol. This continuous need for import and export of metabolites in heterotrophic tissue might explain the high stromule abundance in BY2 cells as well as in non-green tissue like ripening tomato fruits and dark grown seedlings. Furthermore, it could explain why chloroplasts, which generate ATP and NADPH by photosynthetic processes, are reported to show generally lower stromule frequencies than non-green plastids [2]. The fact that chloroplasts develop stromules to a similar extent as epidermal plastids after vacuum infiltration of glucose or sucrose seems to be contradictory at first glance. However, it is well established that under high sugar conditions source activity is suppressed and sink activities are triggered [34]. Naturally, this change occurs during fruit development [35], a process that in tomato fruits goes along with an increase in stromule frequency and length [9]. Furthermore exposure to extracellular glucose and sucrose induces major changes in gene expression [36,37]. Such a change might thus take place also by our sucrose and glucose treatments, since the cycloheximide experiments demonstrate the requirement of *de novo *protein synthesis for stromule induction.

## Conclusions

While up to now only speculations about stromule related processes were possible, the present study provides experimental evidence, which suggests a possible involvement of stromules in carbohydrate metabolism. This supports the idea of stromules being involved in optimizing metabolite exchange. The stromule inducing capacity of glucose and sucrose, important metabolites and signal molecules, provides experimental evidence for the involvement of a typical sugar sensing mechanism in stromule regulation. However, the sugar sensing mechanism and signaling cascades involved remain still unknown and require further investigation. Our model system, the upper leaf epidermis of *Arabidopsis thaliana*, may provide a useful tool for solving these questions.

## Methods

### Chemicals and solutions

All chemicals were purchased from Sigma-Aldrich (Deisenhofen, Germany), Roth (Karlsruhe, Germany), or Serva (Heidelberg, Germany). As buffer for dissolving and diluting sugars and inhibitors, artificial pond water (APW) [38] was used. All solutions were prepared immediately before use.

### Microscopy, hardware and software

For imaging of EGFP fluorescence, an Axioscop 2 upright microscope (Carl Zeiss, Jena, Germany) operating in epifluorescence mode (fluorescence filter 'endowGFP' F41-017 purchased from AHF Analysetechnik, Tübingen, Germany) was used. Images were captured using either an Axiocam HRc camera (Carl Zeiss, Jena, Germany) or a KY-F75 camera (JVC, Japan). Microscope, camera and piezo stepper were controlled by either of the frame grapping software packages AxioVision (Carl Zeiss, Jena, Germany) or DISKUS (Hilgers, Königswinter, Germany).

### Plant material, sample preparation and drug treatments

Transgenic *Arabidopsis thaliana *plants constitutively expressing the chimeric protein FNR/EGFP, which consists of the chloroplast targeting transit peptide of ferredoxin-NADPH-oxidoreductase (FNR) fused to an enhanced derivative of the green fluorescent protein (EGFP), were grown on soil at 120 μEinstein m^-2^s^-1 ^and 60% relative air humidity under a short-day light regime (8 h light/16 h dark). For vacuum infiltration, expanding leaves from 10 - 12 week old plants, which had reached approximately 75% of the size of mature leaves, were harvested. After removing the mid vein, the leaves were cut into four squares and vacuum infiltrated using a 5 ml or 10 ml syringe and a 2 ml reaction tube. The tube was filled with 1.5 ml of the respective solution and a 10 ml syringe was placed on top of the tube. By pulling the plunger of the syringe, vacuum was applied for not longer than 2s. Upon release, the resulting negative pressure in the tissue caused the liquid to flood the intercellular space. The infiltrated leaves were immediately analyzed or further incubated. For treatment of leaf samples with inhibitors of translation, samples were infiltrated with APW supplemented with either 100 μM cycloheximide, 100 μg ml^-1 ^spectinomycin, or 100 μg ml^-1 ^streptomycin and incubated for one hour in darkness. Then the solutions were replaced by APW supplemented with 40 mM sucrose in addition to the respective inhibitor. As a solvent control for cycloheximide treatment, APW was supplemented with DMSO at 0.03%. Each experiment was performed at least three times with leaves of different plants.

### Imaging and data processing

After vacuum infiltration leaf squares were either immediately analyzed (time point 0 h) or incubated at room temperature in the dark for the given time periods (1, 2, or 3 h). For each time point, epidermal plastids of 6 individual leaf sectors were imaged by capturing an image series along the z-axes. The resulting image stack was further processed using the software package AxioVision (Carl Zeiss, Jena, Germany). Image stacks were processed into one 'stacked' 2D image with the help of CombineZP [39] as described previously [40]. After import of the stacked images into the ImageBrowser package (Carl Zeiss, Jena, Germany), plastids with and without stromules were marked following a color code. The resulting image layer, which consisted solely of markings, was exported as an image file. Markings in these images were automatically counted using the Photoshop plug-in FoveaPro 4 (Reindeer Graphics, Asheville, USA). Data files produced with FoveaPro 4 were analyzed with Excel (Microsoft, Redmond, Washington, USA).

### Calculating stromule frequency

The values for SF were calculated as followed. For a time point of a time course experiment image stacks at 6 different spots per leaf square were taken as described (capturing approx. 250 epidermal plastids per spot, i.e. approx. 1500 plastids per leaf square). For each leaf square stromule frequencies of the six spots were calculated resulting in six SF values for each leaf square (for estimating the SF in the palisade parenchyma, only chloroplasts which were completely visible in the taken image stacks were considered). Afterwards SF values were arcsin transformed (arcsin √ SF) according to Waters et al. [9] resulting in stromule index (SI) values. To summarize the data of experimental repeats, for each experiment the arithmetic average and the 99% confidence intervals were calculated using SI values (additional file 1 panel C, E and additional file 3 panel A).

These average SI values and confidence intervals have been converted back into SF values by calculating the square of the sinus of the SI values ((sin SI)^2^) for ease of conveyance. Bar charts of stromule index as well as back-transformed data are shown in additional file 1 panel C-F and additional file 3 panel A and B. For better comparison of the effect of different treatments, the increase or decrease in relation to the initial stromule frequency was plotted in the graphs presented in Figure 2A, D, E and 3A.

## Authors' contributions

MHS designed and carried out all the experiments and wrote the manuscript. RBK participated in the experimental design and helped to draft and write the manuscript. Both authors read and approved the final manuscript.

## Supplementary Material

Additional file 1 **experimental procedure and absolute values of stromule index as well as stromule frequency in epidermal cells**. **A**) Depiction of epidermal cell outlines which illustrates the large variety of cell sizes found in the epidermises of *Arabidopsis thaliana*. Epidermal cells were colored according to the respective size class. Stomata that are shown in gray were not considered. Size bar corresponds to 50 μm. **B**) Schematic depiction of the experimental procedure showing sample preparation, infiltration and data acquisition. **C**) Bar charts showing upper epidermal 'stromule index' mean values for 40 mM sugar (sucrose, sorbitol, mannitol, glucose, or fructose) and buffer control (APW) treatments calculated as described in Material and Methods. Scale maximum of y-axes was set to 1.57, which corresponds to a stromule frequency of 1 (or 100%). Error bars show the 99% confidence intervals and therefore represent the likelihood of the calculated mean value. **D**) By doing the opposite of the mathematical function used for transforming stromule frequencies into 'stromule index', 'stromule index' mean values were back-transformed into stromule frequency values. The same procedure was applied to the 99% confidence intervals. Bar charts showing both values for each time point are depicted in C. To illustrate the relation of stromule frequencies to a 'stromule saturated' tissue, the maxima of the y-axes were set to 1 (or 100%). **E-F**) Absolute stromule indices and back-transformed stromule frequency values for 1 mM, 10 mM and 80 mM sucrose treatments. Click here for file

Additional file 2 **image series for a sucrose induction experiment**. 'Stacked', inverted, gray scaled images of time points 0 h (A), 1 h (B), 2 h (C), 3 h (D) of a 40 mM sucrose induction experiment. Scale bar corresponds to 10 μm. Click here for file

Additional file 3 **absolute values of stromule index as well as stromule frequency in palisade parenchyma cells**. **A and B**) Absolute stromule index and back-transformed stromule frequency values for the 40 mM sorbitol, 40 mM sucrose, 40 mM glucose and APW treatments based on chloroplasts in palisade parenchyma cells. Click here for file
