# Supplementary figures and images for: Induction of stromule formation by extracellular sucrose and glucose in epidermal leaf tissue of Arabidopsis thaliana
Source: BMC Plant Biol. 2011 Aug 16;11:115. doi: 10.1186/1471-2229-11-115 (PMC3167769; doi:10.1186/1471-2229-11-115)

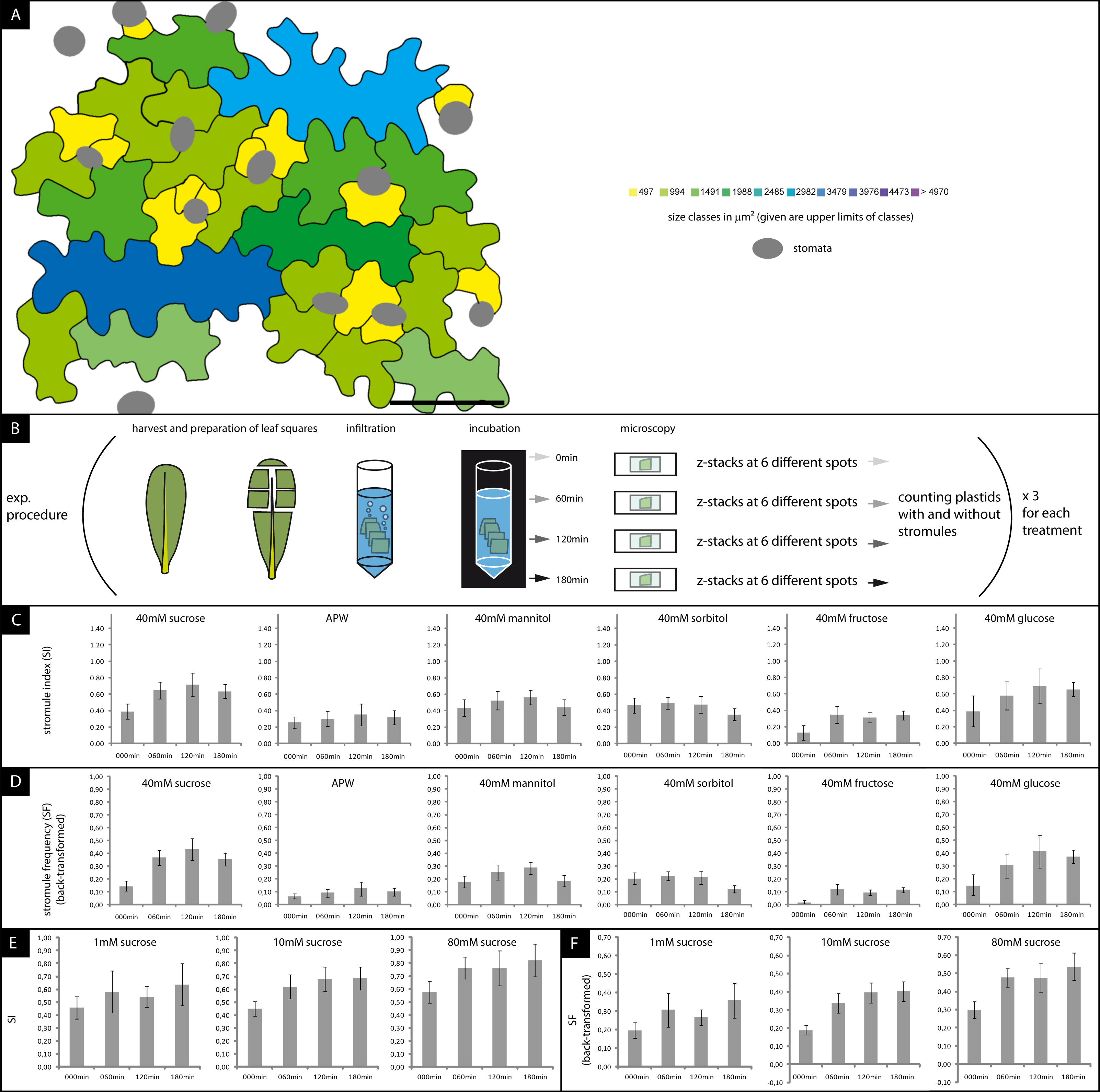

Supplement: Additional file 1 — experimental procedure and absolute values of stromule index as well as stromule frequency in epidermal cells. A) Depiction of epidermal cell outlines which illustrates the large variety of cell sizes found in the epidermises of Arabidopsis thaliana. Epidermal cells were colored according to the respective size class. Stomata that are shown in gray were not considered. Size bar corresponds to 50 μm. B) Schematic depiction of the experimental procedure showing sample preparation, infiltration and data acquisition. C) Bar charts showing upper epidermal 'stromule index' mean values for 40 mM sugar (sucrose, sorbitol, mannitol, glucose, or fructose) and buffer control (APW) treatments calculated as described in Material and Methods. Scale maximum of y-axes was set to 1.57, which corresponds to a stromule frequency of 1 (or 100%). Error bars show the 99% confidence intervals and therefore represent the likelihood of the calculated mean value. D) By doing the opposite of the mathematical function used for transforming stromule frequencies into 'stromule index', 'stromule index' mean values were back-transformed into stromule frequency values. The same procedure was applied to the 99% confidence intervals. Bar charts showing both values for each time point are depicted in C. To illustrate the relation of stromule frequencies to a 'stromule saturated' tissue, the maxima of the y-axes were set to 1 (or 100%). E-F) Absolute stromule indices and back-transformed stromule frequency values for 1 mM, 10 mM and 80 mM sucrose treatments. [file 1471-2229-11-115-S1.TIFF]

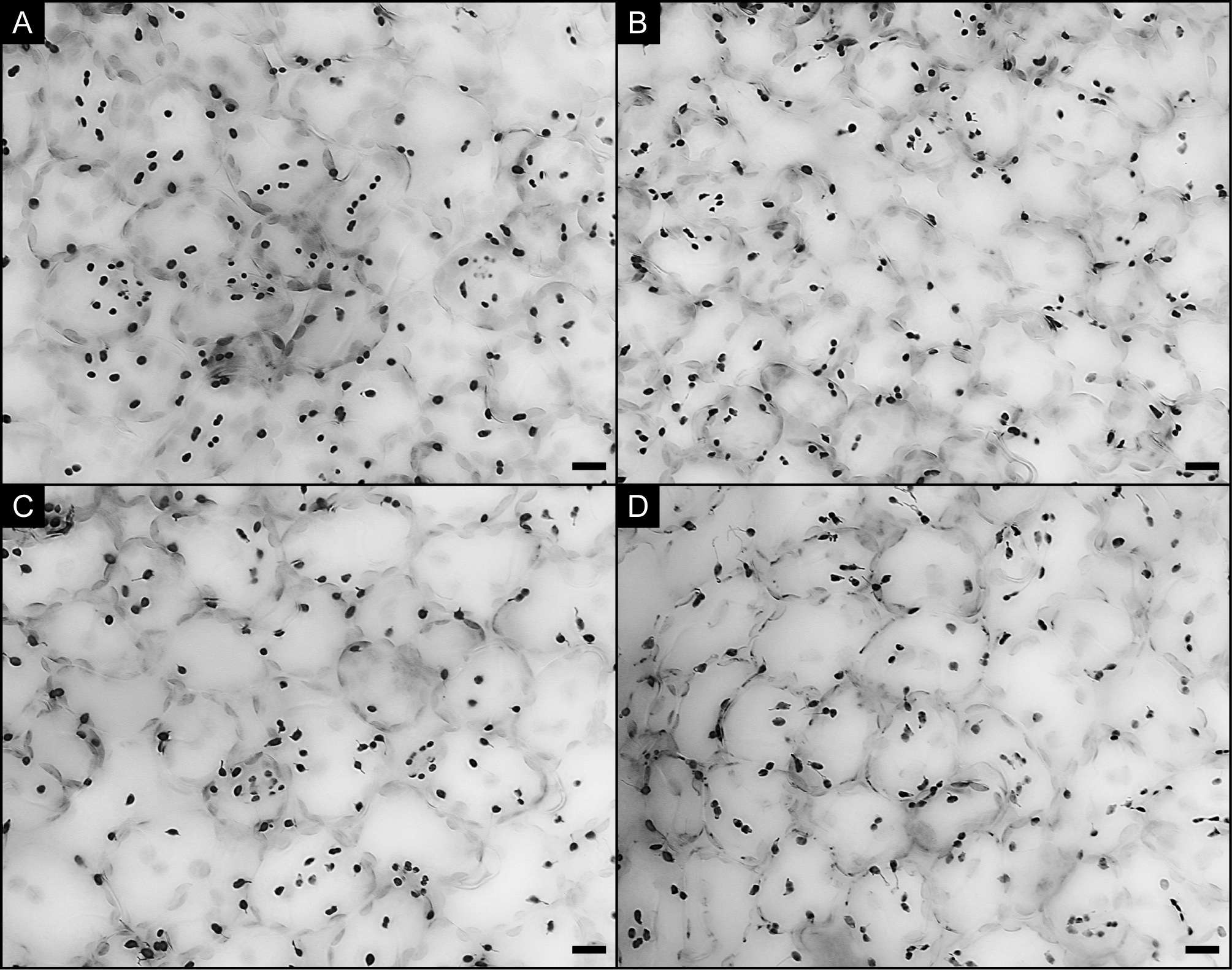

Supplement: Additional file 2 — image series for a sucrose induction experiment. 'Stacked', inverted, gray scaled images of time points 0 h (A), 1 h (B), 2 h (C), 3 h (D) of a 40 mM sucrose induction experiment. Scale bar corresponds to 10 μm. [file 1471-2229-11-115-S2.TIFF]

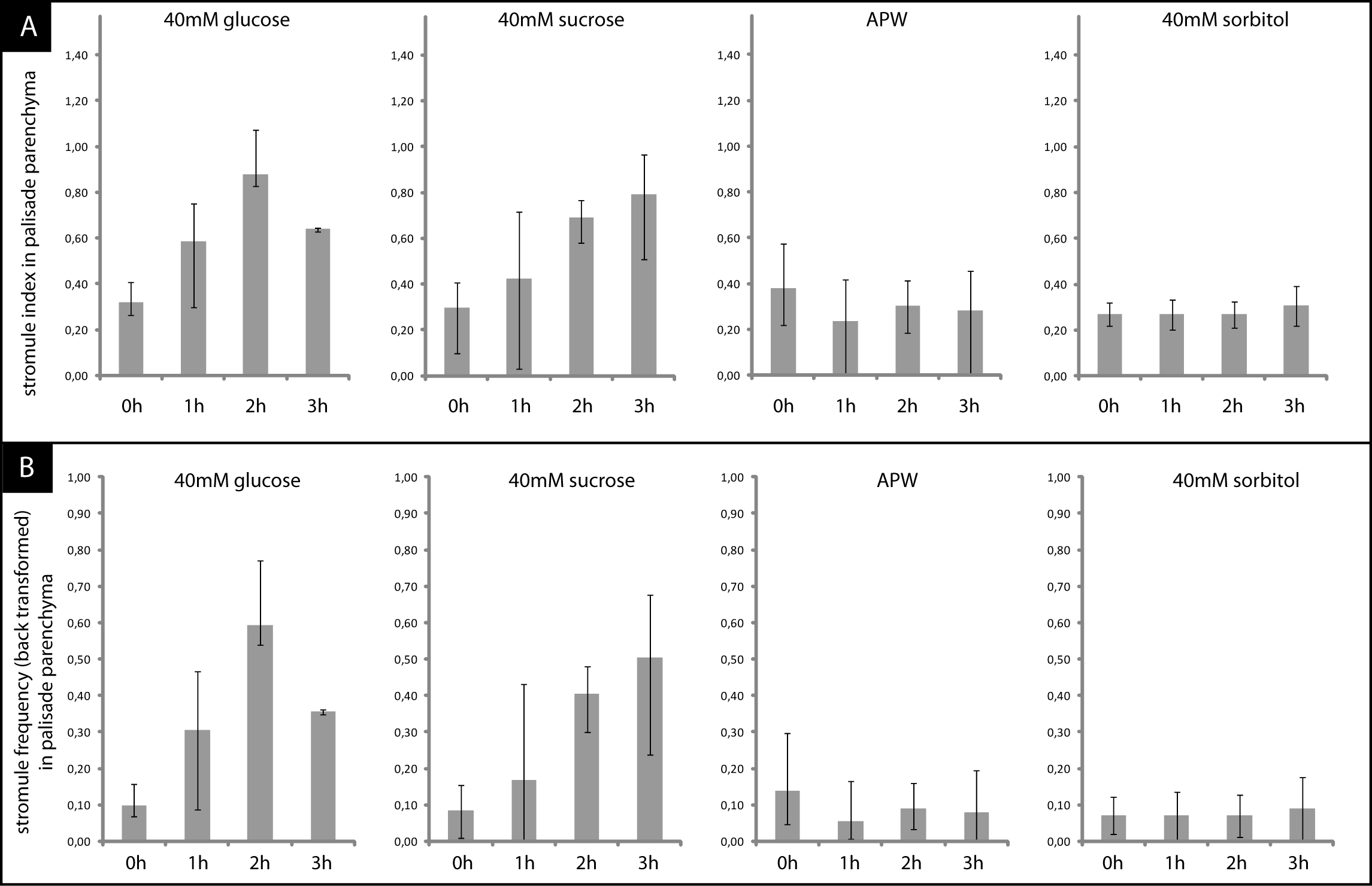

Supplement: Additional file 3 — absolute values of stromule index as well as stromule frequency in palisade parenchyma cells. A and B) Absolute stromule index and back-transformed stromule frequency values for the 40 mM sorbitol, 40 mM sucrose, 40 mM glucose and APW treatments based on chloroplasts in palisade parenchyma cells. [file 1471-2229-11-115-S3.TIFF]
